# Supplementary material for: Content-rich biological network constructed by mining PubMed abstracts
Source: BMC Bioinformatics. 2004 Oct 8;5:147. doi: 10.1186/1471-2105-5-147 (PMC528731; doi:10.1186/1471-2105-5-147)
Supplement: Additional File 5 — The original Chilibot query results of the term "long-term potentiation (LTP)" and 22 other terms, limiting the latest references analyzed to the years 1990, 1995, 2000, and 2004. [file 1471-2105-5-147-S5.bz2 › chilibotAdditionalFile5/ltp2004/html/PLC_ERK.html]

 


 **PLC** and **ERK** 
  
Found 92 abstracts in PubMed,  **30 abstracts were retrieved and analyzed**.  


---

 Search Google  |
 PDF files only 
|  EDU domain only 

---

**Interactive relationship** (e.g. stimulation, inhibition, etc)

- Increased phospho  **ERK**  immunostaining was dependent on  **PLC**  and also on MEK 1 2, an upstream regulator of  **ERK** .  Ref: 12468029 Brain Res, 2002
- The  **PLC**  PKC cascade is required for IL 1beta dependent  **Erk**  and Akt activation their role in proliferation.  Ref: 14612947 Int J Oncol, 2003
- Leukotactin 1 induced  **ERK**  activation is mediated via G i G o protein  **PLC**  PKCdelta Ras cascades in HOS cells.  Ref: 12759139 Life Sci, 2003
- However, TCR induced  **ERK**  activation was suppressed by inhibitors of PKC and  **PLC**  gamma.  Ref: 14680820 Biochem Biophys Res Commun, 2003
- Together, the p43 induced TNF production was controlled by NFkB, p38 MAPK, and  **ERK**  that is dependent on the activities of  **PLC**  and PKC.  Ref: 12543078 Cytokine, 2002
- These results taken together suggest that lysophospholipid receptor activation involves receptor coupling to heterotrimeric Gq subunits with consequent activation of  **PLC** , PKC, and MAPK pathways leading to  **ERK**  phosphorylation.  Ref: 14648542 Glia, 2004
- Taken together, our results suggest that a SHPS 1  **PLC**  gamma complex activate the  **PLC**  PKC cascade, which is required for the activation of IL 1beta dependent  **Erk**  and Akt signalings and cell proliferation.  Ref: 14612947 Int J Oncol, 2003
- We conclude that during hypoxia FBP initiates a series of neuroprotective signals which include  **PLC**  activation, small increases in calcium i, and increased activity of the MEK  **ERK**  signaling pathway.  Ref: 12468029 Brain Res, 2002
- LPA and S1P induced  **ERK**  phosphorylation was dependent on the activation of mitogen activated protein kinase MAPK, phospholipase C  **PLC** , and protein kinase C PKC, but was insensitive to pertussis toxin PTX.  Ref: 14648542 Glia, 2004
- In B cells from Cbl b deficient mice cross linking the BCRs resulted in sustained phosphorylation of Igalpha, Syk, and phospholipase C  **PLC**  gamma2, leading to prolonged calcium mobilization, and increases in extracellular signal regulated kinase  **ERK**  and c Jun NH2 terminal protein kinase JNK phosphorylation and surface expression of the activation marker, CD69.  Ref: 12771181 J Exp Med< MedlineTA>J Exp Med, 2004
- Further, we found that FBP enhancement of phospho  **ERK**  immunostaining depended on calcium i  **PLC**  inhibition and the IP 3 receptor blocker xestospongin C prevented FBP from increasing calcium i and increasing phospho  **ERK**  levels.  Ref: 12468029 Brain Res, 2002
- We propose that the structural resemblance of SC alpha alpha delta 9 to phospholipids allows it to inhibit cellular  **PLC** , thereby providing a possible molecular mechanism for SC alpha alpha delta 9 s effects on oncogenic  **Erk**  activation.  Ref: 12481409 Mol Cancer Ther, 2002
- Inhibition of phospholipase C  **PLC**  resulted in complete inhibition of  **ERK** , while having no affect on p38 activity.  Ref: 14676843 OncogeneOncogene, 2002
- In H ras Q61L transformed Rat 1 fibroblasts with constitutively elevated levels of phosphorylated extracellular signal regulated kinase  **Erk** , SC alpha alpha delta 9 inhibited both proliferation and oncogenic  **Erk**  activation at concentrations that inhibited  **PLC**  in vitro.  Ref: 12481409 Mol Cancer Ther, 2002
- However, a 5 HTR antagonist, MDL 100907, inhibited 5 HT up regulation of  **PLC**  and TGF beta1, while having far less pronounced effects on  **Erk**  1 2.  Ref: 12466135 Am J Pathol, 2002

**Parallel relationship** (e.g. studied together, co-existance, homology, etc.)

- These results suggest that Lkn 1 activates the  **ERK**  pathway by transducing the signal through G i G o protein,  **PLC** , PKCdelta and Ras, and it may play a role for cell proliferation, differentiation, and regulation of gene expression for other cellular processes.  Ref: 12759139 Life Sci, 2003
- In this study, we determined whether FBP s capacity to decrease delayed cell death in hippocampal slice cultures is dependent on  **PLC**  signaling or activation of the intracellular calcium MEK  **ERK**  neuroprotective signaling cascade.  Ref: 12468029 Brain Res, 2002
- Activated TLR2 utilizes PI3 K,  **PLC** , PKC,  **ERK** , and NF kappaB as signaling factors that mediate the oxidative burst of chicken heterophils.  Ref: 14555292 Int Immunopharmacol, 2003
- Pharmacological inhibition of the  **PLC**  PKC cascade by using specific inhibitor for  **PLC**  gamma U73122 and PKC GFX strongly inhibited IL 1beta induced  **Erk**  and Akt activation.  Ref: 14612947 Int J Oncol, 2003
- The phospholipase C  **PLC**  inhibitor, U73122, or the protein kinase C PKC inhibitor, Ro 31 8425, blocked H 2 O 2 induced  **ERK**  phosphorylation.  Ref: 12374624 Free Radic Biol Med, 2002
- Pertussis toxin, an inhibitor of G i G o protein, and phospholipase C  **PLC**  inhibitor blocked Lkn 1 induced activation of  **ERK** .  Ref: 12759139 Life Sci, 2003
- Receptor and kinase activation is linked to distal signaling pathways  **PLC**  gamma1 activation, calcium influx, PKC activation and Ras  **Erk**  activation via the adapter protein LAT Linker for Activation of T cells.  Ref: 14696041 Bioessays, 2004
- In the present study, we used selective pharmacological inhibitors to investigate the roles of phosphatidylinositol 3 kinase PI3 K, phospholipase C  **PLC** , calcium dependent protein kinase C PKC, extra cellular signal regulated kinase  **ERK** , and nuclear translocation factor kappa B NF kappaB on TLR2 mediated oxidative burst.  Ref: 14555292 Int Immunopharmacol, 2003
- The antigen induced tyrosine phosphorylation of FcepsilonRI, Syk, phospholipase C gamma  **PLC**  gamma, activation of c Jun N terminal kinase JNK, extracellular signal regulated kinase  **ERK** , inhibitor of nuclear factor kappaB kinase IKK, and Ca influx were all suppressed in the cells overexpressing Cbl b in the lipid raft.  Ref: 14604964 BloodBlood, 2003
- Finally, transient expression of kinase negative PKCalpha eliminated the 8 OH DPAT evoked block on the H 2 O 2 triggered caspase 3 stimulation, establishing PKCalpha as a link between  **ERK**  and caspase 3 5 HT 1A R > **PLC**  >ERK1 2 >PKCalpha >caspase 3.  Ref: 12676358 Biochim Biophys Acta, 2003
- U 73122 a  **PLC**  inhibitor, wortmannin a PI3 K inhibitor, PD 98059 an  **ERK**  inhibitor, Gö 6976 a PKC inhibitor and Bay 11 7082 a NF kappaB inhibitor significantly decreased LTA stimulated oxidative burst in heterophils by 77%, 30%, 36%, 78%, and 61%, respectively.  Ref: 14555292 Int Immunopharmacol, 2003
